# Towards EMIC rational design: setting the molecular simulation toolbox for enantiopure molecularly imprinted catalysts

**DOI:** 10.1186/s13065-016-0215-7

**Published:** 2016-10-25

**Authors:** Tessa Jalink, Tom Farrand, Carmelo Herdes

**Affiliations:** Department of Chemical Engineering, University of Bath, Bath, BA2 7AY UK

**Keywords:** Racemic mixtures, Stereochemistry, Prochiral substrates, Transition states, Ab-initio simulations, Molecular dynamics, Monte Carlo

## Abstract

A critical appraisal of the current strategies for the synthesis of enantiopure drugs is presented, along with a systematic background for the computational design of stereoselective porous polymers. These materials aim to achieve the enantiomeric excess of any chiral drug, avoiding the racemic separation. Particular emphasis is given to link statistical mechanics methods to the description of each one of the experimental stages within the catalyst’s synthesis, setting a framework for the fundamental study of the emerging field of molecularly imprinted catalysts.

**Graphical abstract:**
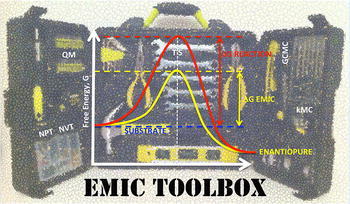
The envisaged modelling tools in the EMIC toolbox: quantum mechanics (QM), molecular dynamics and Monte Carlo (in the NPT and NVT ensembles), grand canonical Monte Carlo (GCMC) and kinetic Monte Carlo (kMC), for the synthesis of an enantiopure drug via our proposed EMIC catalyst.

The envisaged modelling tools in the EMIC toolbox: quantum mechanics (QM), molecular dynamics and Monte Carlo (in the NPT and NVT ensembles), grand canonical Monte Carlo (GCMC) and kinetic Monte Carlo (kMC), for the synthesis of an enantiopure drug via our proposed EMIC catalyst.

## Background

Nature as a whole is a chiral system, many of the molecules that constitute living organisms are chiral and, in the vast majority of cases, preference is shown for one of the enantiomers. For example, proteins are formed exclusively of the L form of amino acids. Meanwhile, saccharide units of the D form singularly constitute carbohydrates; in the same manner, enantiomeric forms in the building blocks of DNA and RNA (d-ribose or d-deoxyribose) have been observed [1].

Enantiomers have the same physical properties with the exception that they interact differently with polarised light. Regarding the chemical properties, both enantiomers solely differ in their reactivity with other chiral molecules. Hence, a chiral molecule only manifests itself as such by the influence of polarised light or other chiral molecules.

Biological systems, such as proteins and enzymes that catalyse life’s essential reactions have a three-dimensional structure and establish preferences to interact with one of the enantiomers of other molecules. The effect of these interactions is the basis for the study of chiral drugs. As a result of their chirality, racemic drugs can have different effects on our bodies. There are chiral drugs in which each one of the enantiomers could produce opposite effects in the organism, in other cases, the effect is similar, but one of the enantiomers is more active than the other (eutomer and distomer, respectively). While in some cases, one enantiomer is active and the other is inactive and also can occur that one enantiomer has a beneficial effect meanwhile the other is toxic.

Through an evolutionary pathway, nature has become stereoselective, being capable of synthesising *the best for a purpose* of the enantiomers. A practically endless list of chiral compounds provided by nature can be compiled. The tobacco leaves only produce the levorotatory *S*-nicotine. The coca only makes *S*-cocaine. The sugarcane generates d-sucrose exclusively. Limonene is an interesting case, which implies that genetic information drives the biosynthesis of the enantiomers, the dextrorotatory d-limonene is found in the orange or lemon peel. Meanwhile, in the mint, it is found as the levorotatory l-limonene and in the turpentine (derived from pines) as the racemic mixture (±)-limonene [2].

Chiral drugs dominate the modern pharmaceutical landscape, making up to 40–50% of the market in 2013 with 9 of the top 10 bestseller drugs being chiral [3]. These drugs are sold as racemic mixtures or as a single enantiomer. Currently, there is a significant trend in the pharmaceutical industry to produce what is called “chiral switches”: chiral drugs already commercialised as racemates that could be developed as a single enantiomer [4]. The idea behind these chiral switches is the fact that the enantiomers exhibit different behaviour when they are exposed to the chiral environment that is the human body. This discrimination between enantiomers—or chiral recognition—depends on the degree of interaction that each enantiomer exhibits with the chiral binding site in the body.

Pointing out the enantioselective action of chiral drugs at the beginning of modern pharmacology was regarded as vain within the global profile of drug activity. Nowadays, this is no longer the case. At this very moment, most of the existing patents for drugs consisting of racemic mixtures are coming to an end and the race to obtain new ones for enantiopure production has already begun [5].

Therefore, there is a need for systematic studies to enhance the understanding of eutomers and to guide their stereoselective synthesis. This work introduces the most relevant molecular simulation methods to help in the design of enantiopure molecularly imprinted catalysts, EMICs. A well-designed EMIC would create a considerable impact in the way the synthesis of enantiopure drugs is performed. An EMIC could circumvent the effort involved in separating racemic mixtures and enable direct access to the eutomer, which in turn reduces the necessary dosage and the chronic side effects of the racemate as well as simplifying dosage-effect studies.

The description of different molecular simulation techniques for the study and development of these efficient catalysts throughout their synthesis stages is the principal purpose of this contribution and the central pillar of our on-going research efforts, translating the principles of enzyme catalysis to the design of EMICs from a molecular perspective.

### Currents paths from racemate to enantiopure drugs

The separation of racemates into their enantiomers is a difficult task, e.g. distillation cannot be employed, as both enantiomers will have the same bubble point. To achieve an enantiopure separation the technique used must discriminate based on the stereo orientation of the enantiomer. The most relevant categories of chiral drugs and the current ways to obtain the enantiopure ones follow.

There are three categories that all chiral drugs fall under [6].

- Most chiral drugs have one key bioactive enantiomer. In this case, one of the enantiomers, the eutomer, is much more active and efficient than the other. The distomer can either be less active, toxic or produce undesirable effects. Drugs that fall under this category will often benefit from the synthesis of an enantiopure drug, e.g. ethambutol, whereas the (S,S)-(+)-enantiomer is used to treat tuberculosis, the (R,R)-(−)-ethambutol causes blindness [7].
- Some chiral drugs have equally bioactive enantiomers. Here, the two enantiomers would have the same activity and identical pharmacodynamic properties. There are only a few chiral medications that may fall under this category, but none has been confirmed [6].
- Finally, some chiral drugs can undergo chiral inversion in the body. These drugs have the unique property that the eutomer or distomer can be converted into the other by our body. For these drugs, it can be unnecessary to develop a single enantiomer drug. For example, in the case of ibuprofen, while the racemic mixture is 50/50 when administered, some distomers are converted into eutomers in the body, ultimately making the drug more potent [8].

There are six main ways to obtain enantiopure drugs from either racemic mixtures or substrates [9].

- Synthesis of diastereomeric salts by treatment with an enantiomer. The salts of the two enantiomers have different solubilities, allowing them to be separated from each other.
- Utilising the various reaction rates of the two enantiomers with the addition of a different enantiopure compound. Up to 50% of the enantiomer that reacts more slowly can be recovered from the racemate.
- Other resolution of racemates also takes advantage of the differing reaction rates to separate the mixture. However, the unrecovered enantiomer is converted back into a racemic mixture. This process is then repeated until a higher yield of the eutomer is recovered.
- Some approaches take advantage of naturally occurring enantiopure compounds. The natural enantiopure compound is modified to create the desired enantiopure drug. This method is extremely useful when the product you want has a similar chemical structure to the naturally occurring enantiomer and is used in such cases.
- Synthesis of the enantiopure compound from prochiral substrates by the introduction of a chiral auxiliary to the racemic substrate mixture to separate the two enantiomers. The auxiliary is then removed post-separation. This method is effective, but the auxiliary is required in a stoichiometric quantity. Because of this, the auxiliary must be cheap and easy to produce.
- Selective adsorption, a stereoselective adsorbent is used to remove only one enantiomer thoroughly from the racemate. This method has a large advantage over using an auxiliary because sub-stoichiometric amounts of the adsorbent can be used (and re-used) for an adequate separation. Current separation techniques include the use of enzymes and homogeneous chiral metal containing complexes.

Here, we propose EMICs as a seventh alternative to obtaining the eutomer avoiding the racemic separation. Such a catalyst could be done by exploiting on the field of molecularly imprinted polymers (MIPs) [10], the basic concepts of molecular imprinting can be adopted to create a catalytic polymer network that will promote the transition state (TS) of a particular reaction in a lock and key fashion.

### Following nature’s example

Natural enzymes possess an arrangement of functional groups responsible for their specificity [11, 12]. The substrate-enzyme binding interactions are rather complex and consist of a combination of electrostatic interactions, hydrogen bonds, hydrophobic interactions, and other contributions. Then, some prerequisites have to be fulfilled for the preparation of a material showing enzyme-like catalytic activity towards the eutomer, i.e. to construct an EMIC.

First, a cavity has to be made with a defined shape. This shape can correspond to the substrate or, even better, to the TS of the reaction. Due to the TS instability a transition state analogue, TSA, must be found. The cavity can also adopt the shape of the eutomer. Functional groups have to be introduced to act as binding sites within the cavity in a defined stereochemistry. These requirements were introduced with the imprinting protocol conceptualised by Dickey [13] and implemented by Wulff [14] and Mosbach [15].

**Fig. 1:**
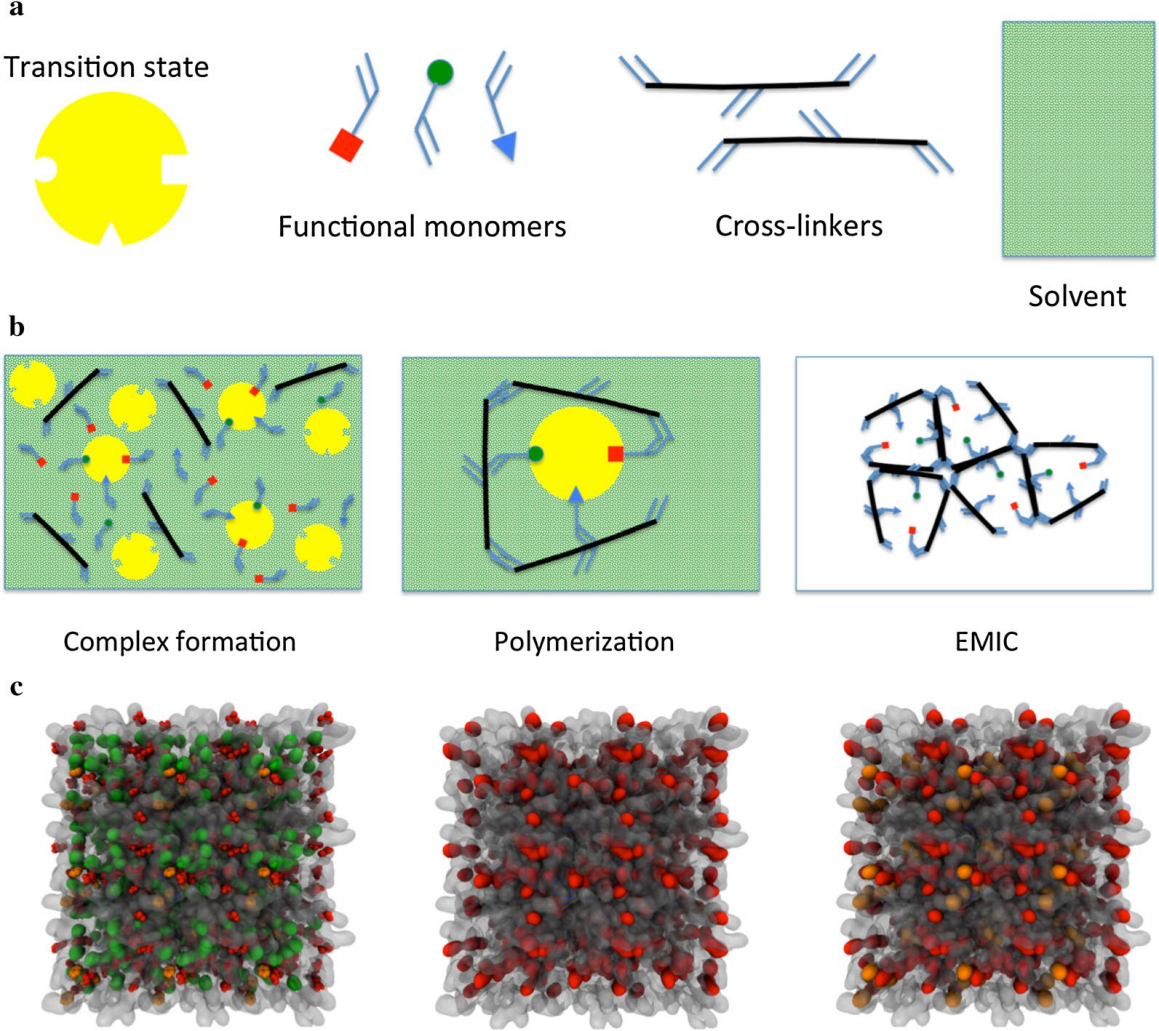
**a** Main EMIC components. **b** Synthesis stages. **c** Computer graphics visualizations of three stages for a pyridine selective polymer: *left* final configuration of the equilibrium mixture of the functional monomer methacrylic acid (*red*), cross-linker ethylene glycol dimethacrylate (*white*), solvent chloroform (*green*) and template pyridine (*orange*); *centre* same configuration with the solvent and template removed; *right* pyridine molecules rebinding sites. Model details can be found elsewhere [29]

The schematic and components of the imprinting protocol via TS can be seen in Fig. 1a, b. The polymerisable functional groups are usually bound by covalent or non-covalent interaction to the TS. This complex is then copolymerized in the presence of large amounts of cross-linking agent and inert solvent (the latter acting as a porogen). After removal of the TS, an imprint containing functional groups in a certain orientation remains in the highly cross-linked polymer. The shape of the imprint and the arrangement of the functional groups are complementary to the structure of the TS. This procedure furnishes porous polymers with a permanent pore structure and a high inner surface area, where the preferred binding for the TS lowers the activation energy of the desired reaction and has thus a catalytic effect on the reaction rate. This concept was already postulated by Pauling [16] and later discussed more in detail by Jencks [17]. The concept was shown to be correct by Lerner [18] and by Schultz [19], independently, by generating antibodies against a stable TSA of a reaction.

Following the technique described above, recent years have seen remarkable progress in the design of molecularly imprinted catalysts [20]. Numerous reviews on the molecular imprinting procedure have been published [21–27]. However, a comparison between these catalysts and enzymes [22], shows that enzymes are still in every case several orders of magnitude catalytically more efficient, but in a few cases, the efforts have reached the activity of catalytic antibodies, e.g., in the hydrolysis of carbamates [28].

### What do modelling techniques have to offer for the design of better MICs and EMICs?

The binding site homogeneity in enzymes is high, whereas MICs, have a broad distribution of activity, and there is no method available at the moment to reduce this broadness significantly. Though some progress has been made in the preparation of MICs, for modest use in industry and wider application in research, refining the experimental imprinting procedures with insights gained from statistical mechanics tools could make further developments.

Improvement of the mass transfer in the imprinted networks, reduction of the polyclonality of cavities, an increase of available active sites (in particular with the frequent noncovalent interaction) and development of further suitable groupings for catalysis are just some problems at the forefront of investigations [20, 22]. Some researchers have concluded that a larger extent of self-assembly can result in a higher specificity. Others have claimed that the shape of the imprinted cavity is the main aspect of molecular recognition and that a change in the form will result in a lower level of identification. More recently, more and more researchers tend to support a modest extent of self-assembly as the condition for the strongest molecular recognition. Undoubtedly, the design of MICs is attracting an extensive research effort [20].

The idea behind EMICs is clear and straightforward, but the huge pool of variables in its synthesis and characterization requires some rational screening strategies. We believe these strategies could evolve from simultaneous and synergic use of modelling tools with experimental work for the sound design of EMICs in silico. All the variables involved in the synthesis can be independently controlled, and their impact systematically assessed to prepare better catalysts. With molecular models, we seek to understand how imprinted materials are created and what happens to TSAs and substrate molecules in these imprinted cavities to become eutomers. This will help to elucidate the different contributions of each parameter to the overall catalytic effect with the use of proper control systems, for the ultimate developments of better MICs for various reactions and EMICs for specific diseases. Figure 1c, obtained through our molecular dynamics (MD) methodology [29], shows the complexation, polymerization and cavity rebinding points for a pyridine-selective polymer.

Some detailed atomistic simulations have been employed for the computational design of imprinted polymers [30–34]. Recently, a more general approach derives a set of design principles and backs up the possibility of efficiently imprinting drugs [35], although very few specific examples of molecular modelling efforts could be found for MICs design [20]. While some interesting insights have been gained [30–34], most of these efforts suffer from two significant drawbacks. First, they focus on a single cavity (neglecting issues related to the heterogeneity of binding sites and porosity). Second, the material optimisation is reduced to a simplified scoring function based on the internal energy of complexation, rather than on proper adsorption or rebinding isotherms or reaction yield as measured in experiments.

We should aim to develop models and methodologies that feature a sufficient level of realism and detail, specifically based on accurate force fields, and that reflect some underlying principles behind the materials formation and function. These protocols should imitate the actual process of MICs formation, characterization and applications within four stages of development:

Stage 1 involves a mixture of TSA (or substrate or product), functional monomers and cross-linkers. *Ab initio* calculations are envisaged to identify the plausible TSA of the desired reaction, obtain the partial charge distributions of these structures, and describe the complex TSA cavity-regarding binding site energy. The equilibrium properties of the mixture (TSA, functional monomer, cross-linker and solvent) could be obtained by molecular dynamics [29] or Monte Carlo [35] approaches to mimic the synthesis conditions in NPT and NVT ensembles.

In Stage 2, the polymerization of functional monomers and cross-linkers should be modelled from the equilibrated mixture structure (a direct outcome of Stage 1). The idea is to focus on the generation of the functional/selective catalytic cavity-ignoring, for a while, the details of the network formation. However, the explicit account of the new bonds formed during the polymerization step can be attained by kinetic Monte Carlo [36].

In Stage 3, the imprint TSA is removed. The model can be further extended to imitate some post-formational modifications, such as cavity shrinking and introduction of defects. This resulting structure would serve as a porous matrix for both the structural characterization and applications. The resulting model EMIC could be used as the material structure for adsorption studies. The grand canonical Monte Carlo (GCMC) method [37] is appropriate to describe the re-binding behaviour of the EMIC under study.

In Stage 4, before the reaction, the TSA is bound to the EMIC in a pre-equilibrium step. The bound TSA is converted under catalysis of the TSA-EMIC to the product and is then released. At this final stage, the different reaction kinetics (regarding rates of reactions of different orders of magnitude expected for the diffusion and binding of the template to the polymer) can be investigated by using the probability-weighted dynamic Monte Carlo method [38]. Complex molecular geometries may require the employment of advanced techniques such as configurational bias Monte Carlo [39] and cavity/energy bias Monte Carlo [40] to efficiently explore the binding sites.

As briefly described above, required modelling tools for EMIC’s rational design protocol (models and methods) are available, but so far these tools remain unrelated to the field. The compilation of such a computational toolbox would encompass linking various pieces of research together in a consistent workflow, standardising inputs and outputs between the stages and methods. Many aspects of the sketched protocol are challenging and require substantial expertise in the areas of molecular modelling, programming and statistical mechanics. However, the expected outcomes in the understanding of these systems are worth the effort.

The largest gain of the proposed theoretical approach to these systems is that it will allow going beyond current knowledge and exploring these novel formulations. However, the validation of any computer simulation strategy requires the comparison with nature; i.e. the model must be able to reproduce the essential properties of a system that has been already explored experimentally. An excellent source for test cases is the first book in the area of MICs with its substantial amount of experimental work and applications [20].

## Concluding remarks

This toolbox could be very useful in improving the scope and applicability of MICs for more advanced catalysis, as the EMICs proposed here, (i.e. the selective catalysis of enantiopure drugs). The fundamental efforts of the described tasks would help to ask, and hopefully to answer, the “what if” questions for a range of possible catalytic systems, focusing on the in silico performance rank of the candidate materials, bypassing the economic constraints of such search through real experiments. As a result, EMICs could be synthesised to corroborate, or dispute, the predictions and guide the ultimately necessary experimental work.

For instance, one can compare the re-binding affinity of a synthesised EMIC using the TSA, against the theoretical adsorption affinity of that EMIC but imprinted with the TS. Such a comparison would serve as a characterization approach and pre-screening of plausible EMIC formulations. This type of study will be experimentally inconclusive, due to the instability associated with the TS. However, this useful exercise will be setting a theoretical limit to help identify the best TSA for a specified system. The presented computational techniques allow us to fulfil both characterization processes (i.e. the structural and the energetic ones) using an entirely controlled framework.

MICs are easy to prepare and handle, and the EMICs will inherit these qualities. MICs can be prepared in large quantities by suspension polymerization, and stable particles of uniform diameter can be easily obtained [20]. In addition to beads or broken particles, MICs can also be prepared in other very different forms, such as monoliths, microcapsules, membranes or surfaces [10]. MICs have both excellent mechanical and thermal stability. Frequently, they can be used for a long time in a continuous process, or they can be reused many times. As a result of their insolubility, they can be easily filtered off after a reaction, or they can be placed in a flow reactor. Whereas enzymes and antibodies degrade under harsh conditions such as high temperature, chemically aggressive media, and high and low pH, MICs show better behaviour in most cases, and they can be applied directly in chemical processes since they are rather stable materials.
